# Supplementary material for: Impact of COVID-19 on mortality in coastal Kenya: a longitudinal open cohort study
Source: Nat Commun. 2023 Oct 28;14:6879. doi: 10.1038/s41467-023-42615-6 (PMC10613220; doi:10.1038/s41467-023-42615-6)
Supplement: Supplementary file 1 — Supplementary Information [file 41467_2023_42615_MOESM1_ESM.pdf]

Supplementary Materials for

Impact of COVID-19 on mortality in coastal Kenya: a longitudinal open cohort study

Authors: M. Otiende, A. Nyaguara, C. Bottomley, D. Walumbe, G. Mochamah, D. Amadi, C. Nyundo, E. W. Kagucia, A. O. Etyang, I. M. O. Adetifa, S. P. C. Brand, E. Maitha, E. Chondo, E. Nzomo, R. Aman, M. Mwangangi, P. Amoth, K. Kasera, W. Ng'ang'a, E. Barasa, B. Tsofa, J. Mwangangi, P. Bejon, A. Agweyu, T. N. Williams, J. A. G. Scott

Correspondence to: motiende@kemri-wellcome.org

This PDF file includes:

Supplementary Text
Figs. S1 to S10
Tables S1 to S8
References

Supplementary Text

Statistical models

Equation S1: Negative binomial model for mortality

$$y_t \backsim \text{Negative Binomial}\ (\mu_t, \phi),$$

$$\log \mu_t = \beta_0 + \beta_1 t + \beta_2 \cos\left(\frac{2\pi t}{12}\right) + \beta_3 \sin\left(\frac{2\pi t}{12}\right) + \log \gamma_t.$$

$$y_t = \text{no. of deaths per month}$$

The model includes terms to account for log-linear trend and seasonality (sine and cosine terms) and an offset ($\log \gamma_t$) to account for changes in person years of observation.

Analysis of air temperature

To improve the prediction of all-cause mortality during the pandemic, we considered the potential link between temperature and mortality[1]. We used air temperature data (for the area covered by the KHDSS) at 2 meters height from the Copernicus ERA5 global weather and climate reanalysis dataset[2]. This dataset contains temperature estimates modelled from data collected using satellites and ground monitors. We extracted the air temperature data into R using the ecwmfr package[3] and included the monthly average temperature as a covariate in the regression model.

Temperature data for the area covered by the KHDSS suggest a very narrow band of temporal variation in temperature which is unlikely to impact mortality and confound our analyses (Figure S4). This was confirmed on testing where the models with and without the temperature covariate fit the data equally well and we selected the simpler model. Excess mortality summaries based on the model with the temperature covariate are shown in Table S4. The estimates were similar to those based on the model without temperature (Table 1 and Table 2). The KHDSS area, spanning 900 square kilometres, experiences a uniform climate and we therefore do not expect any spatial variation in temperatures.

Data Quality Checks

Because there is a theoretical possibility of under-ascertainment of deaths during the period 23rd March 2020 to 25th October 2020, when Kilifi HDSS field operations were suspended, we performed two data quality checks to investigate this possibility.

(i) Reporter accuracy

If there is no household member available to interview during a re-enumeration round, the interview may be conducted with a respondent from a neighbouring household. One possibility that might lead to under-ascertainment of deaths during the pandemic period is that the fieldwork during this period relied more heavily on external respondents who may not have known about recent deaths.

To examine this possibility we looked at the proportion of reports given by a respondent who was a resident of the same household. Table S5 illustrates the variation in this proportion over the 13-year period of the mortality analysis. Deaths were reported by a member of the same household within a range of 71-83% in the period before fieldwork was suspended by Government restrictions on 23rd March 2020. After fieldwork resumed on October 26th there was no evidence of an increase in external witnesses; in fact, the reporting of deaths was more likely to originate from household members than during the baseline period, probably because of reduced mobility and a greater likelihood of finding a respondent at home.

(ii) Under-estimation of person years of observation during the lockdown

To examine the impact of the pandemic on our denominators, we plotted monthly PYO by age group between 2010 and 2022 (Figure S5). Among infants the figures show a substantial drop in observed person years that accumulates from the beginning of 2020, reaches a nadir towards the end of the same year, recovering during 2021. We believe this reflects our inability to detect new births during the 7-month period when field activities were curtailed due to pandemic restrictions. Deaths occurring among children who were not captured by the HDSS fieldwork would not appear in the numerator of mortality rates. In addition, we excluded mortality among children aged <12 months so these very marked fluctuations in PYO do not have any impact on the results of the excess mortality analyses.

Within the age groups 1-4 years, 5-14 years and 15-44 years we observe two phenomena: (i) the number of PYO rises over the early part of the baseline period but is attenuated in the last 5 years maintaining a plateau; note, this plateau is not evident among older persons; (ii) there is a dip in PYO in 2020 which reverses during 2021 in a similar pattern, but at a much smaller magnitude, to the changes observed in infants. We believe this is also an artefact of the fieldwork methodology based on the dates assigned to mortality risk at the observation of migration events. When an existing resident migrates out of the HDSS area we observe this, in retrospect, at a household visit, and assign the end of the resident's risk period as the date he or she migrated out as reported by the household respondent. By contrast, when a new resident migrates into the HDSS, although we collect the date of in-migration, we assign the beginning of the resident's risk period as the first date we documented the

period of residence, which is the date of the household visit. The HDSS therefore systematically underestimates the total number of individuals actually residing within homesteads located in the catchment area. This underestimation is necessary to maintain correspondence between the numerator (deaths) and the denominator (person years at risk); individuals who migrated in and died before they were captured in a re-enumeration round would not be observed and therefore back-dating the PYO to the date of arrival for all in-migrants, rather than the date of first observation of a living resident, would result in' immortal time'.

The degree to which the HDSS underestimates the actual population living in the catchment area is a function of the average interval between the date of in-migration and the date of observation of the in-migration event. If this is prolonged, as occurred during the pandemic, when field activities were suspended for 7 months, it is inevitable that the sum PYO will decline. As this inter-date interval shortened with the resumption of the normal fieldwork cycle, the PYO returned to baseline. It is important to note that, whilst the PYO declined temporarily during the lockdown, the correspondence between numerator and denominator was retained as whilst the PYO of some in-migrants was missing, any deaths occurring in this group was also unobserved.

Because there are chance fluctuations in the total PYO from year to year we would normally estimate deaths (and predict future deaths) based on a modelled estimate of the PYO. However, given the unpredictable impact of the pandemic lockdown on the observation of PYO we have estimated mortality rates and predicted future mortality rates on the bases of observed, not modelled PYO.

(iii) Migrating home to die

Although we have argued that the correspondence between deaths and person years of observation remained intact during the pandemic, there is still a possibility that the unobserved PYO and unobserved deaths among migrants may have biased the mortality estimates during the pandemic. This could arise if the migrants experienced atypical mortality risks, for example, when a sick person returns to his or her rural home to die, a phenomenon known as the unhealthy in-migrant. In normal circumstances the return of these sick individuals contributes to all-cause mortality in Kilifi HDSS but during the pandemic they may not have been able to travel back to Kilifi or they may have travelled and died before they could be observed. If this effect was substantial, the unhealthy in-migrant deaths missed during the pandemic period may have masked an excess of COVID-19 related deaths among stable residents of Kilifi HDSS. (Obviously, this also applies to Kilifi HDSS residents who originate from elsewhere and may wish to return to their distant birthplace when sick or dying. However, the effect is likely to be geographically asymmetrical because Kilifi, a poor rural area, experiences substantial net out-

migration among young and middle aged adults, who migrate to the cities looking for work.)

To explore this potential bias we calculated the 7-month risk of death in a cohort of residents selected on 23rd March 2020, the day after the first case of COVID-19 was detected in Kilifi, and compared this cohort to similar cohorts selected on the same date in previous years. Table S6 shows the annual mortality risks. Figure S6 shows the survival of each cohort from 2010-2020. These analyses illustrate the mortality risk and survival function after excluding any in-migrants during the risk period. The results do not suggest an excess of mortality risk or reduced survival in 2020/21 among stable residents of the Kilifi HDSS.

Supplementary Figures

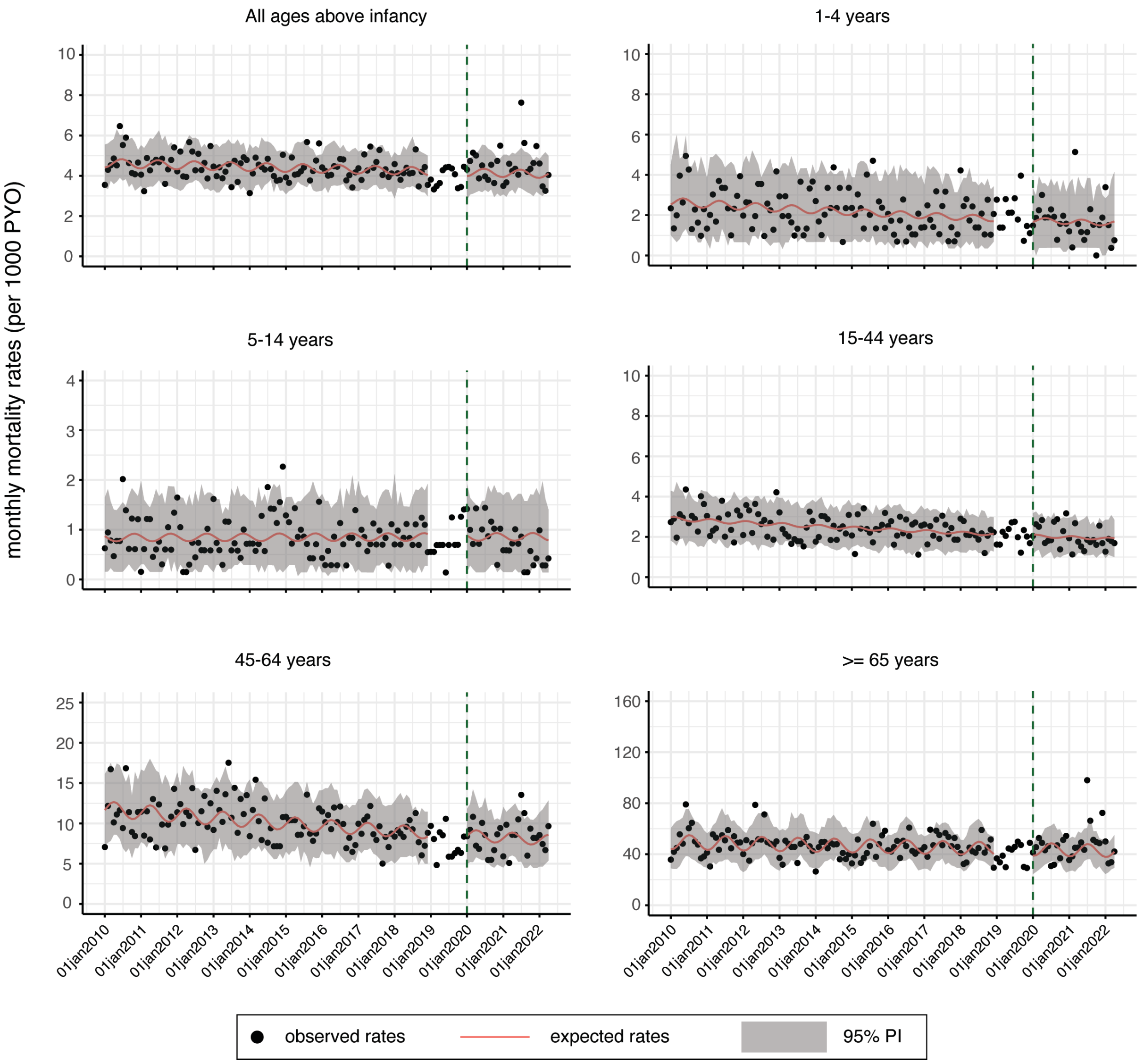

Figure S1. **Observed and expected monthly death rates from 1st January 2010 – 30th April 2022.** Expected deaths rates were estimated from a negative binomial regression fitted using baseline data from 2010 to 2018. The year 2019 was excluded from the baseline model because, on internal validation analyses, there was a significant deficit in all-cause mortality in this year. This deficit was followed by a significant excess in mortality in the first 3 months of 2020 suggesting a temporal delay in the deaths expected for 2019. This is unlikely to be related to the pandemic because neither SARS-CoV-2 infection not the pandemic response were established in Kilifi HDSS until the end of March 2020.

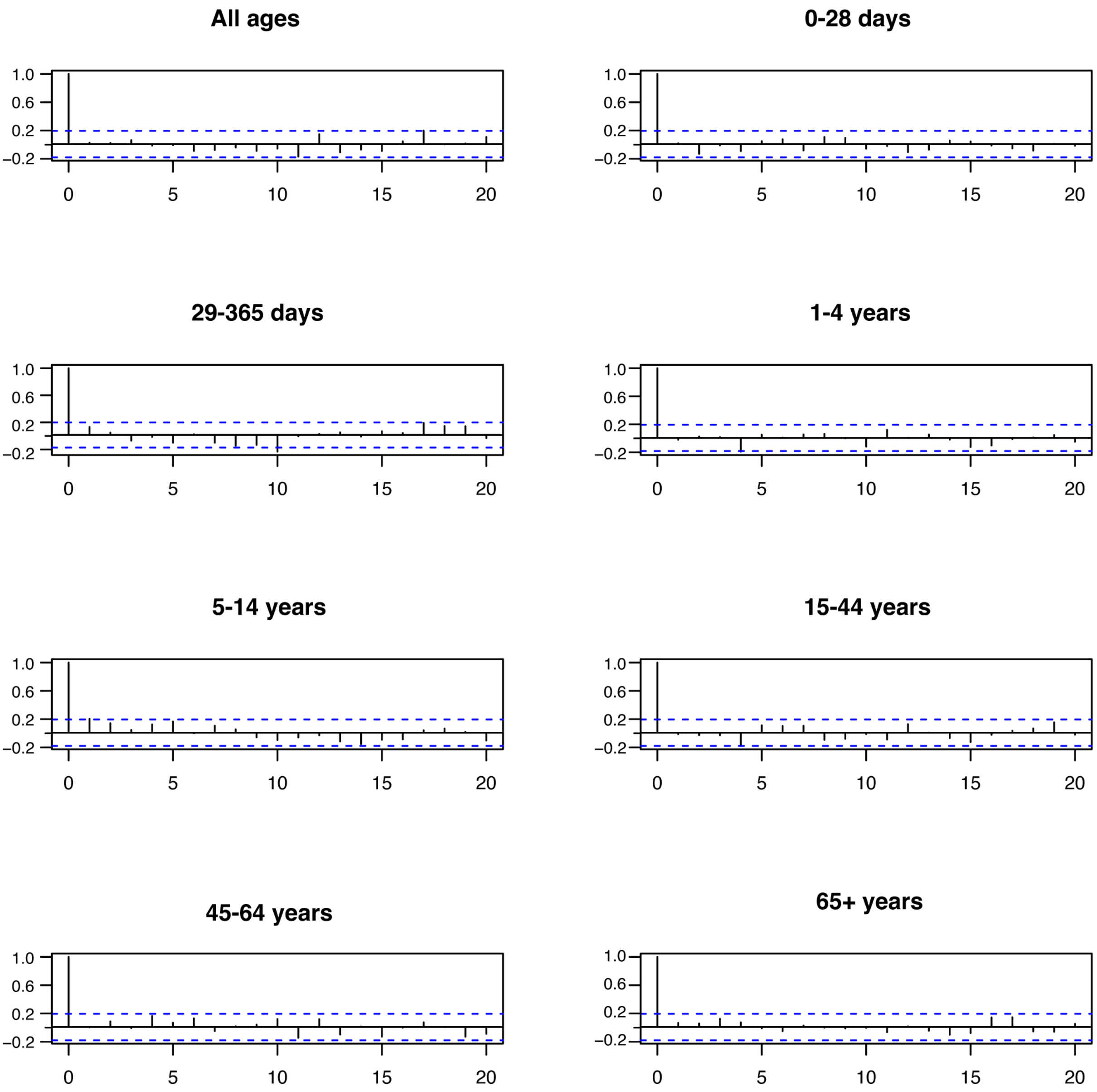

Figure S2. **Age-specific autocorrelation functions (ACF) of baseline monthly mortality rates.** If any spike of lag ≥1 is outside the bounds (blue dashed lines) or if substantially 5% of the spikes are outside these bounds, then this indicates the presence of autocorrelation.

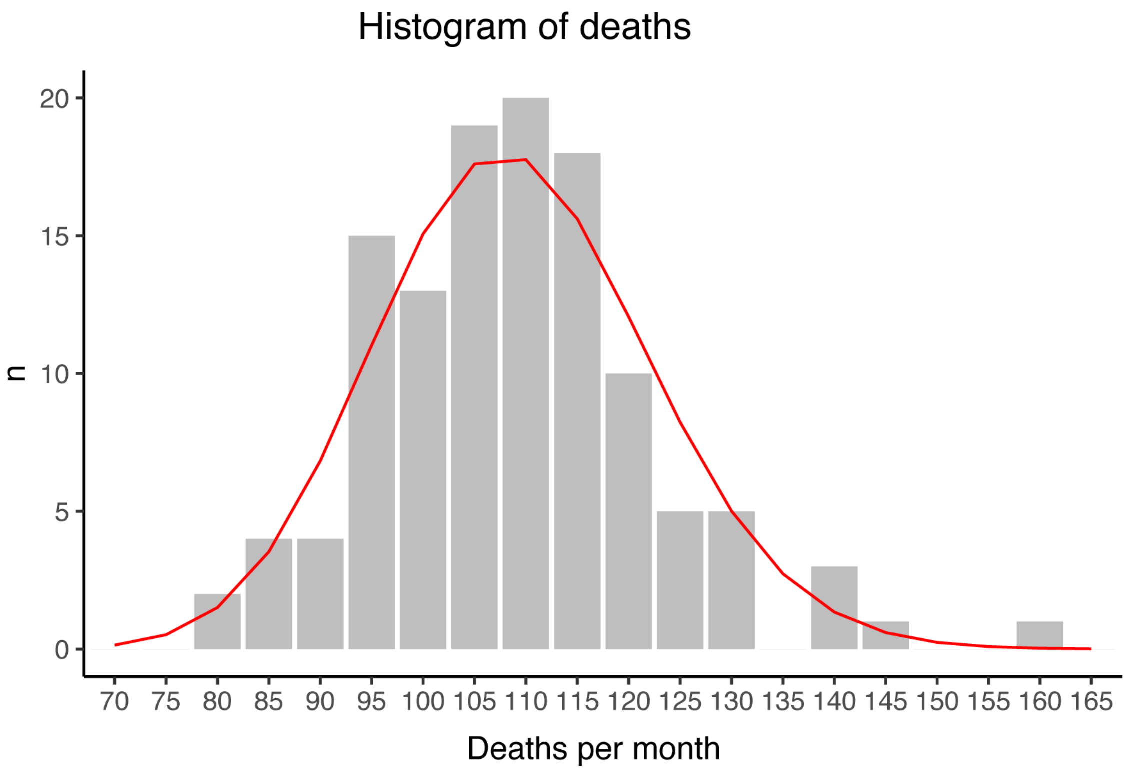

Figure S3. **Negative binomial distribution (red curve) superimposed on the empirical distribution of monthly death counts (grey bars) in the baseline period (2010-2018).** We compared the distribution of the observed monthly death counts to the predicted distribution from the negative binomial model. The predicted distribution is similar to the observed distribution indicating that the model is a suitable fit for the data.

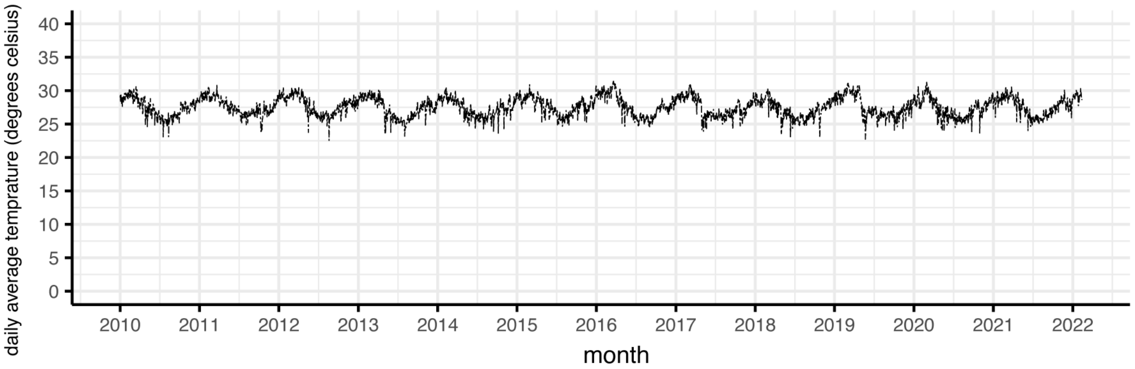

Figure S4. **Average daily temperatures (degrees Celsius) from January 2010 to February 2022 within the KHDSS area.** Data was obtained from the fifth generation ECMWF (European Centre for Medium-Range Weather Forecasts) reanalysis - ERA5, using coordinate specifications that cover the KHDSS area. Kilifi has a relatively stable temperature throughout the year. Including this temperature covariate in our model did not improve the model fit.

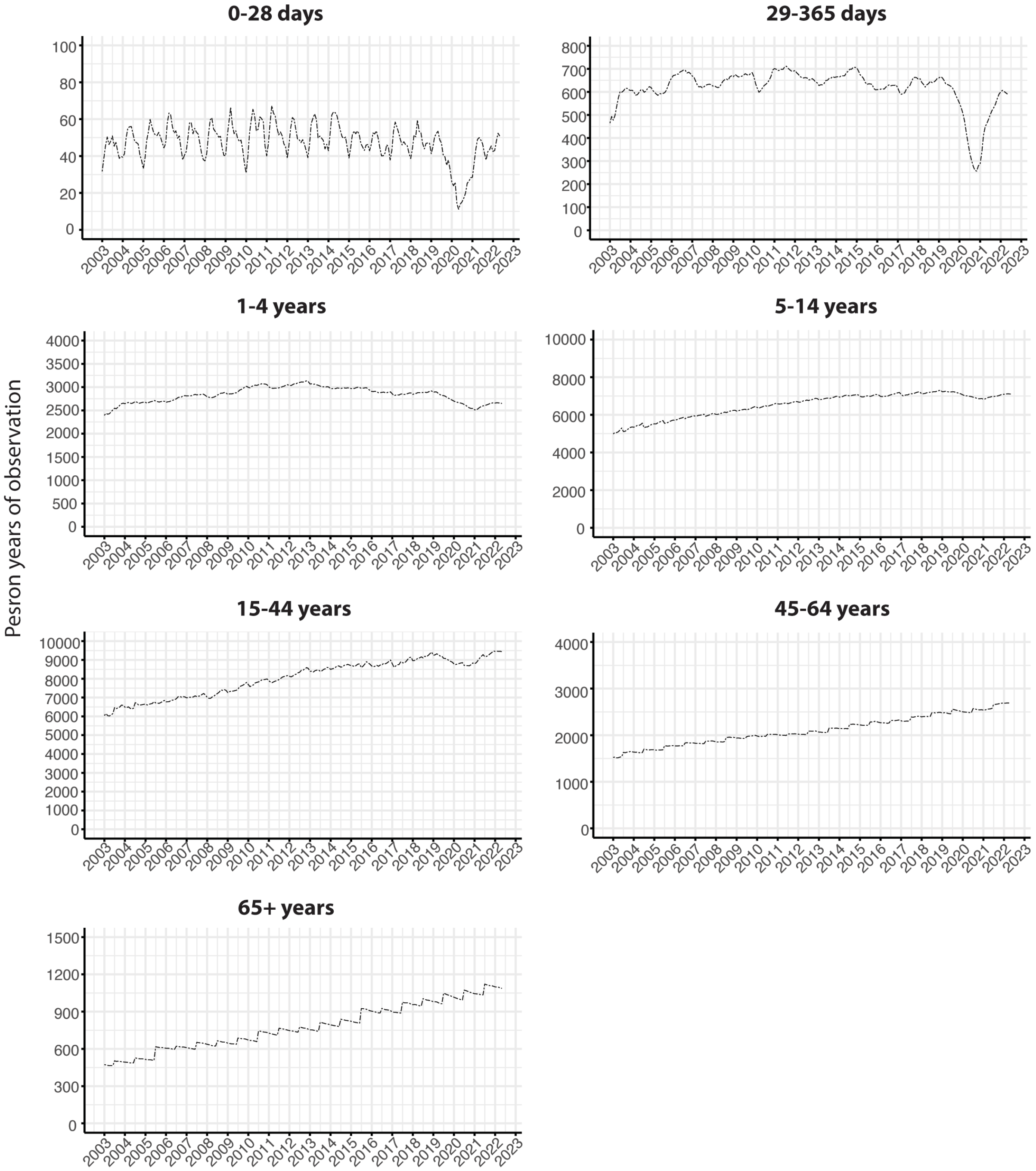

Figure S5. **Trends of empiric person years of observation by age within Kilifi HDSS**

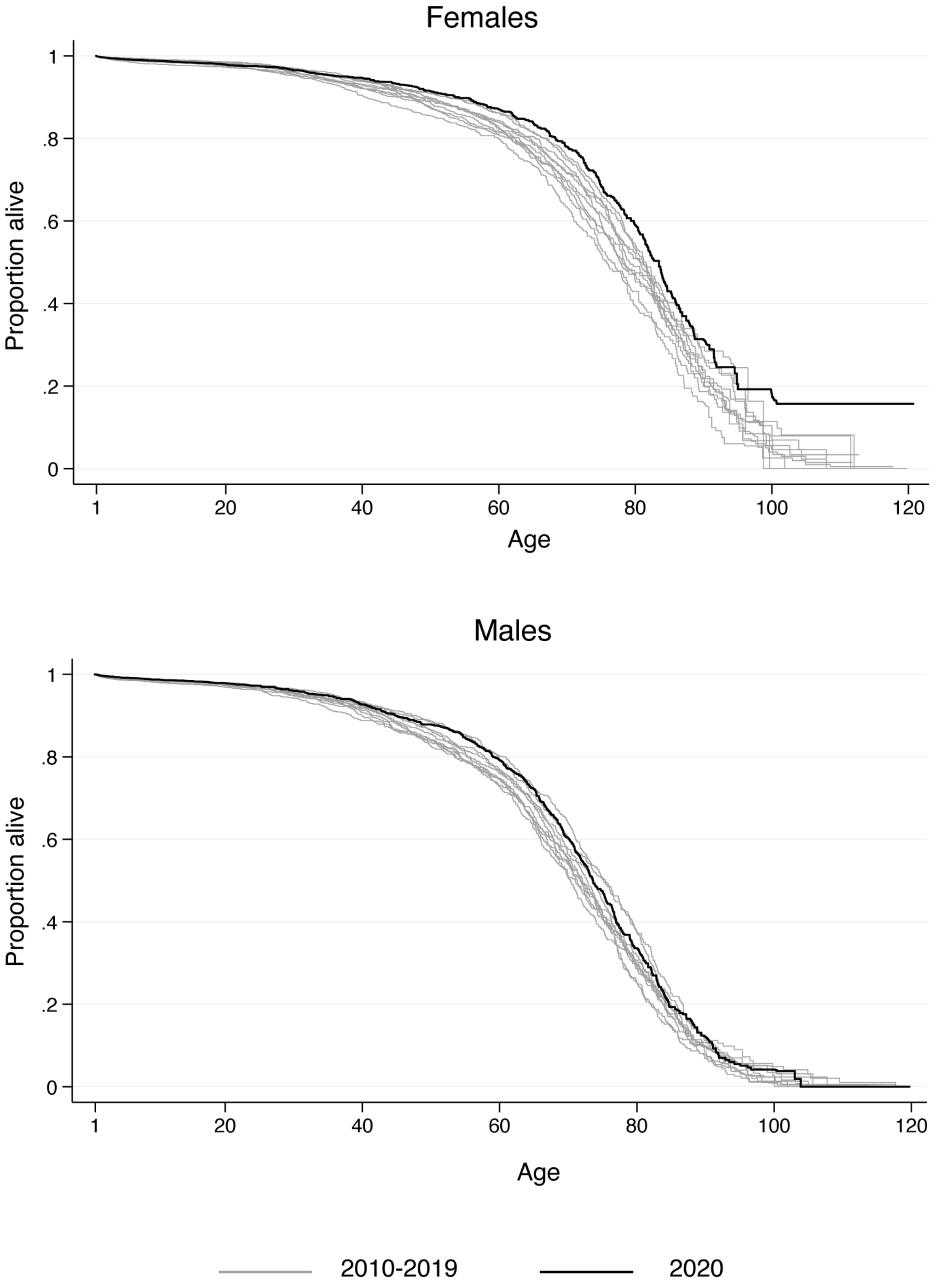

Figure S6. **Annual period survival curves for cohorts resident on the 23rd March each year from 2010-2020 for males and females.** Survival time is age, and the survival function starts at age 1 year, for consistency with other analyses. The light grey lines are individual survival curves of cohorts for the years 2010-2019 and the black solid lines are the survival curves for the 2020 cohort.

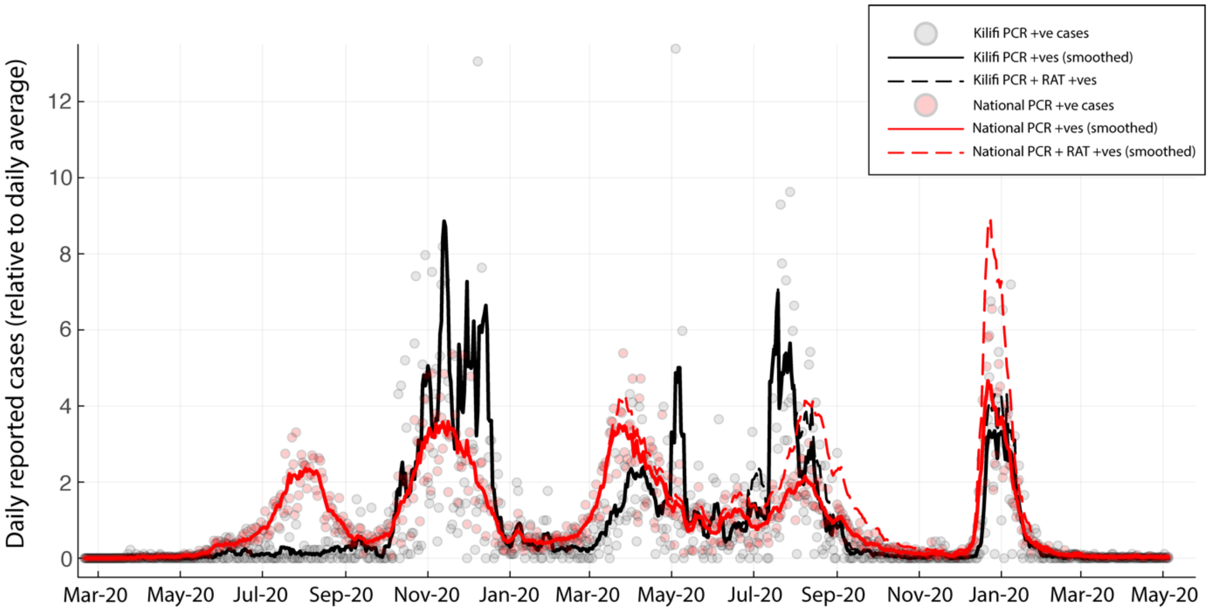

Figure S7. **Ratio of COVID-19 cases reported daily to the mean daily number in National COVID-19 surveillance and the subset of data from Kilifi County alone.** Initially, cases were defined by PCR testing alone. After 5th March 2021, rapid antigen testing was introduced into the surveillance system.

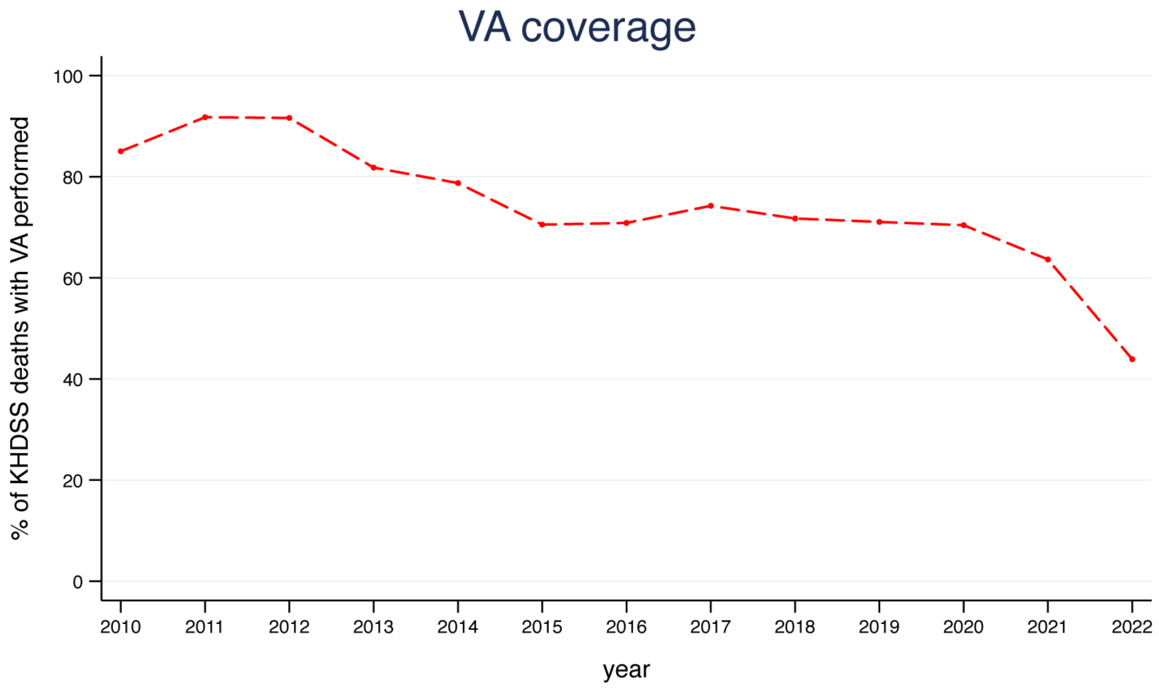

Figure S8. **The proportion of all deaths recorded in Kilifi Health and Demographic Surveillance System that are investigated by verbal autopsy during the baseline period 2010-2019.** Between 1st January 2020 and 31st December 2021, 1822 (66%) of 2736 deaths were investigated by verbal autopsy. Reasons for incomplete investigation include; inappropriate respondent, respondent not at home, postponed interview or refusal by the respondent. Despite longer intervals between death and ascertainment of death in 2020/21 finding an appropriate respondent was only marginally more difficult

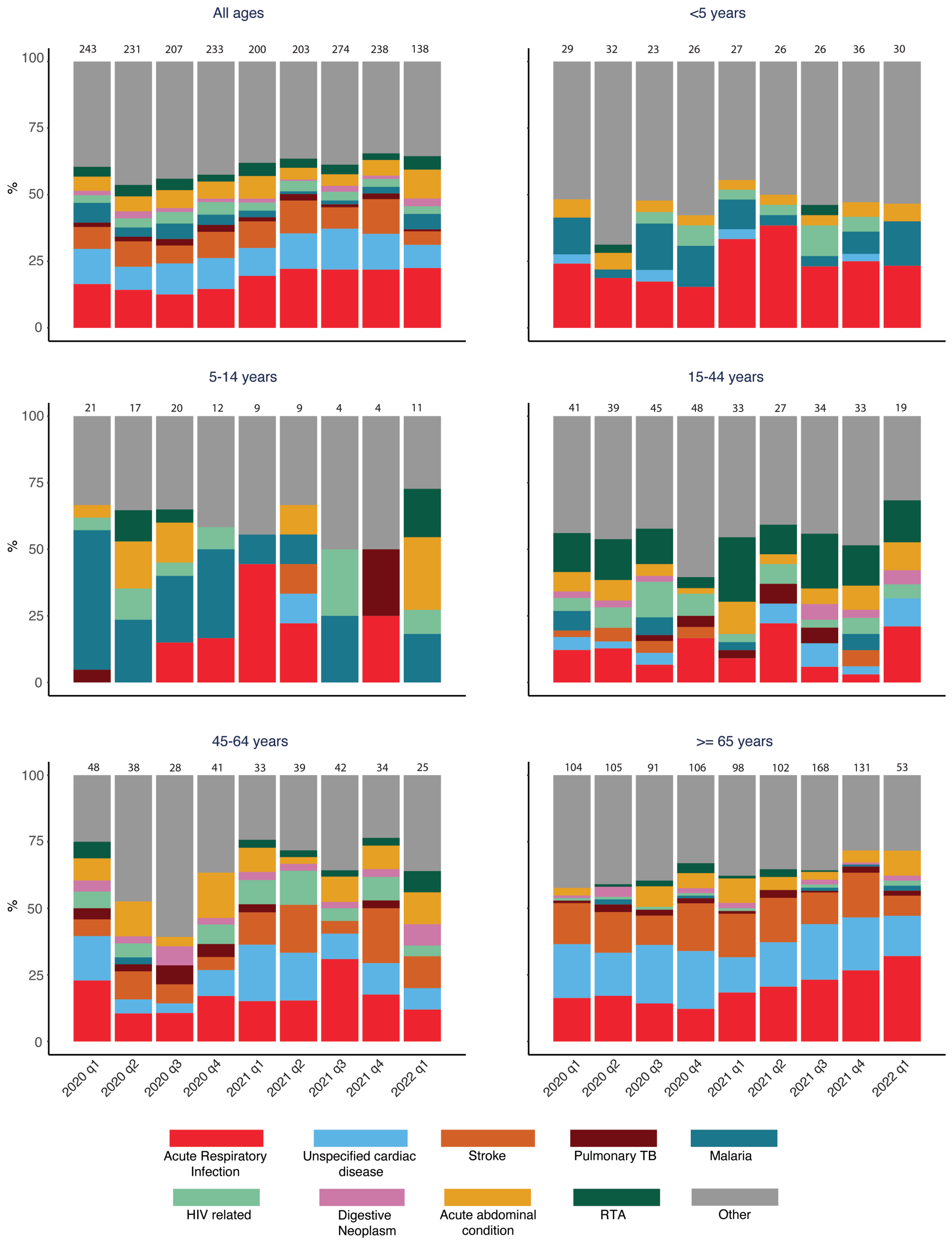

Figure S9. **Quarterly cause-specific mortality fractions by Verbal Autopsy from January 2020 to March 2022.**

Physician Review classification	Probable COVID-19	Possible COVID-19	Unlikely to be COVID-19
Physician Review results	N=20	N=9	N=74
COVID-19 algorithm probability > 89%	n=15	n=4	n=12

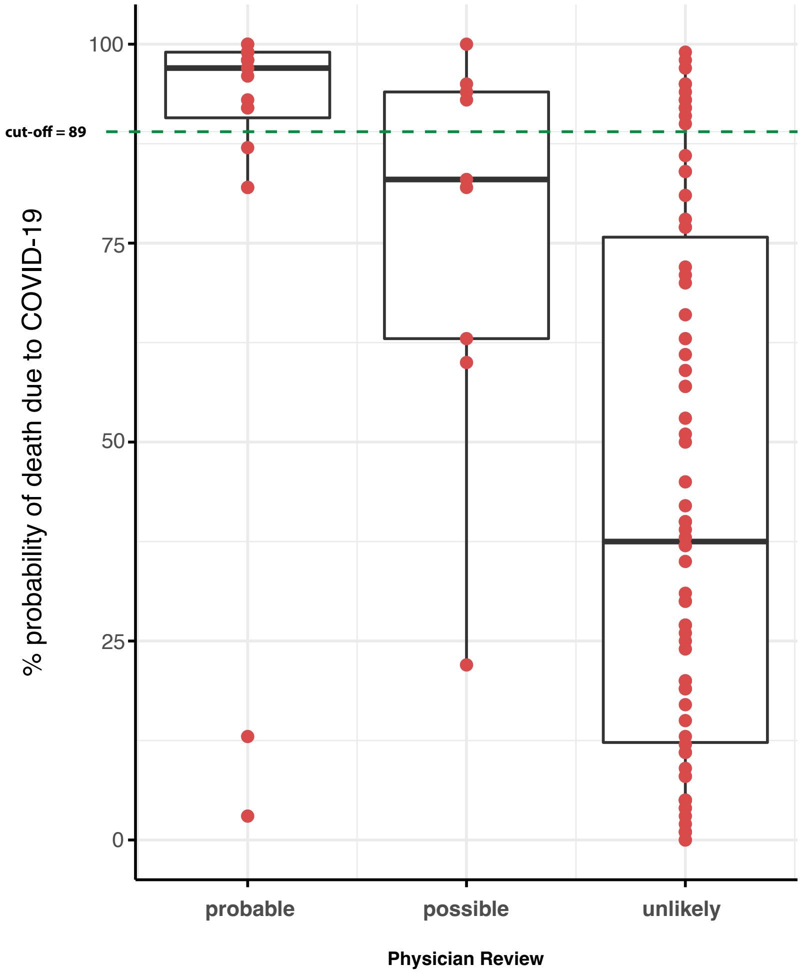

Figure S10. **Verbal Autopsy Assignment of COVID-19 as cause of death by CRMS algorithm and by physician review.** Red dots are the probability values from the CRMS algorithm. They have been overlaid on their respective box plots which shows their distribution. The plots have been categorized according to the physician review categories. The green horizontal dotted line shows the probability cut-off value (89%) beyond which a death is classified as COVID-19 related by the CRMS algorithm[4].

Supplementary Tables

Table S1a. **Excess deaths in waves 1-5, and from January 1, 2020, to December 31, 2021 in Kilifi HDSS residents aged >1 year – female deaths only.**

Age	Deaths		Excess mortality			
group	observed	expected	N	%	95% PI	Rate/100,000
1st April 2020 – 16th April 2022 (Waves 1-5)						
1-4y	49	42	7	16.7	-8.1, 70.5	22.2
5-14y	56	56	0	0.0	-22.5, 48.3	0.0
15-44y	195	212	-17	-8.0	-19.3, 8.2	-14.5
45-64y	244	219	25	11.4	1.3, 26.8	65.9
≥65y	620	533	87	16.3	6.4, 27.0	552.8
All ages*	1164	1062	102	9.6	3.6, 16.6	35.5
1st January 2020 – 31st December 2021						
1-4y	50	41	9	22.0	-5.7, 75.6	29.1
5-14y	58	55	3	5.5	-22.2, 45.3	3.7
15-44y	197	208	-11	-5.3	-15.1, 11.0	-9.6
45-64y	241	215	26	12.1	-0.2, 28.3	70.7
≥65y	606	518	88	17.0	6.5, 29.1	579.3
All ages*	1152	1037	115	11.1	4.8, 19.3	41.2

*All ages excluding infants <1 year old

Table S1b. **Excess deaths in waves 1-5, and from January 1, 2020 to December 31, 2021 in Kilifi HDSS residents aged >1 year – male deaths only.**

Age group	Deaths observed	Deaths expected	Excess mortality N	%	95% PI	Rate/100,000
1st April 2020 – 16th April 2022 (Waves 1-5)						
1-4y	58	65	-7	-10.8	-29.6, 23.3	-21.6
5-14y	65	90	-25	-27.8	-38.8, -4.9	-28.8
15-44y	242	226	16	7.1	-3.7, 23.0	15.3
45-64y	281	304	-23	-7.6	-18.3, 3.5	-89.5
≥65y	637	599	38	6.3	-1.2, 17.3	365.1
All ages*	1283	1284	-1	-0.1	-5.6, 6.2	-0.4
1st January 2020 – 31st December 2021						
1-4y	57	64	-7	-10.9	-29.2, 21.5	-22.1
5-14y	72	88	-16	-18.2	-29.1, 7.6	-18.9
15-44y	245	220	25	11.4	0.6, 26.3	24.8
45-64y	278	296	-18	-6.1	-17.4, 6.1	-72.4
≥65y	637	584	53	9.1	1.3, 20.0	524.3
All ages*	1289	1252	37	3	-2.7, 9.1	14.7

*All ages excluding infants <1 year old

Table S2a. **Excess deaths from 1st January 2020 to 16th April 2022 in Kilifi HDSS residents aged ≥1y, pre-pandemic and by wave analysis period – female deaths only.**

Age	Deaths		Excess mortality			
group	observed	expected	N	%	95% PI	Rate/100,000
1st January 2020 – 31st March 2020 (pre-pandemic period)						
1-4y	9	6	3	50.0	-20.9, 350.0	75.6
5-14y	7	7	0	0.0	-46.2, 133.3	0.0
15-44y	31	27	4	14.8	-15.1, 88.3	28.4
45-64y	28	27	1	3.7	-27.3, 60.4	22.4
≥65y	69	58	11	19.0	-9.2, 48.5	607.8
All ages*	144	125	19	15.2	-0.8, 30.1	54.7
1st April 2020 – 4th October 2020 (Wave 1 - wild type)						
1-4y	12	11	1	9.1	-28.4, 134.4	12.6
5-14y	25	14	11	78.6	6.8, 303.9	52.4
15-44y	60	53	7	13.2	-13.5, 60.9	24.5
45-64y	53	58	-5	-8.6	-26.7, 22.1	-54.1
≥65y	120	136	-16	-11.8	-22.6, 1.4	-423.2
All ages*	270	272	-2	-0.7	-10.7, 9.7	-2.8
5th October 2020 – 14th February 2021 (Wave 2 - wild type)						
1-4y	10	7	3	42.9	-18.7, 229.4	54.7
5-14y	9	11	-2	-18.2	-41.9, 78.6	-13.6
15-44y	32	37	-5	-13.5	-30.9, 26.3	-24.6
45-64y	39	36	3	8.3	-18.9, 52.1	45.2
≥65y	91	90	1	1.1	-14.4, 23.5	36.4
All ages*	181	181	0	0.0	-14.3, 13.6	0.0
15th February 2021 – 4th June 2021 (Wave 3 – Beta-Alpha)						
1-4y	12	6	6	100.0	10.9, 513.7	130.9
5-14y	7	7	0	0.0	-43.5, 390.5	0.0
15-44y	27	32	-5	-15.6	-39.0, 27.0	-28.7
45-64y	34	34	0	0.0	-22.3, 36.1	0.0
≥65y	93	75	18	24.0	2.8, 54.2	794.1
All ages*	173	154	19	12.3	-2.3, 27.3	45.0
5th June 2021 – 11th December 2021 (Wave 4 - Delta)						
1-4y	7	10	-3	-30.0	-57.8, 60.7	-37.3
5-14y	9	15	-6	-40.0	-61.3, 27.4	-28.0
15-44y	44	54	-10	-18.5	-36.7, 18.7	-32.8
45-64y	77	54	23	42.6	11.2, 85.2	233.5
≥65y	214	146	68	46.6	26.1, 67.1	1636.2
All ages*	351	279	72	25.8	14.9, 35.9	97.3
12th December 2021 – 16th April 2022 (Wave 5 – Omicron BA1)						
1-4y	8	7	1	14.3	-30.0, 174.8	18.2
5-14y	6	9	-3	-33.3	-63.3, 67.3	-20.5
15-44y	32	37	-5	-13.5	-33.5, 28.1	-24.0
45-64y	41	37	4	10.8	-18.4, 56.4	59.9
≥65y	102	87	15	17.2	0.1, 40.5	538.0
All ages*	189	177	12	6.8	-7.0, 17.6	23.8

*All ages excluding infants <1 year old.

Table S2b. **Excess deaths from 1st January 2020 to 16th April 2022 in Kilifi HDSS residents aged ≥1y, pre-pandemic and by wave analysis period – male deaths only.**

Age	Deaths		Excess mortality			
**group**	**observed**	**expected**	**N**	**%**	**95% PI**	**Rate/100,000**
1st January 2020 – 31st March 2020 (pre-pandemic period)						
1-4y	6	8	-2	-25.0	-57.1, 50.0	-48.9
5-14y	15	11	4	36.4	-16.7, 200.0	37.4
15-44y	32	29	3	10.3	-22.0, 64.4	24.5
45-64y	43	39	4	10.3	-14.9, 72.6	132.4
≥65y	72	63	9	14.3	-11.7, 52.4	736.6
All ages*	168	150	18	12.0	-6.7, 27.0	57.5
1st April 2020 – 4th October 2020 (Wave 1 - wild type)						
1-4y	19	18	1	5.6	-27.5, 69.8	12.3
5-14y	21	21	0	0.0	-30.2, 67.9	0.0
15-44y	64	54	10	18.5	-8.5, 60.0	39.8
45-64y	58	74	-16	-21.6	-38.5, 1.2	-256.5
≥65y	137	161	-24	-14.9	-24.8, 0.4	-940.1
All ages*	299	328	-29	-8.8	-19.8, 2.1	-45.5
5th October 2020 – 14th February 2021 (Wave 2 - wild type)						
1-4y	7	11	-4	-36.4	-56.1, 49.9	-71.5
5-14y	11	17	-6	-35.3	-57.6, 25.0	-39.4
15-44y	47	40	7	17.5	-7.7, 74.6	38.7
45-64y	49	53	-4	-7.5	-24.3, 22.5	-89.2
≥65y	109	97	12	12.4	-4.5, 40.9	650.7
All ages*	223	218	5	2.3	-10.2, 16.4	11.1
15th February 2021 – 4th June 2021 (Wave 3 – Beta-Alpha)						
1-4y	8	10	-2	-20.0	-47.4, 82.3	-42.6
5-14y	14	13	1	7.7	-27.2, 201.8	7.8
15-44y	34	34	0	0.0	-26.6, 42.8	0.0
45-64y	41	47	-6	-12.8	-31.8, 13.8	-160.1
≥65y	86	85	1	1.2	-17.8, 29.9	66.3
All ages*	183	189	-6	-3.2	-15.5, 9.4	-15.7
5th June 2021 – 11th December 2021 (Wave 4 - Delta)						
1-4y	13	16	-3	-18.8	-47.9, 49.8	-36.2
5-14y	10	23	-13	-56.5	-69.7, -37.1	-58.7
15-44y	58	56	2	3.6	-11.9, 40.5	7.4
45-64y	79	75	4	5.3	-15.7, 37.9	59.9
≥65y	200	164	36	22.0	9.0, 39.0	1332.3
All ages*	360	334	26	7.8	-1.2, 17.4	38.8
12th December 2021 – 16th April 2022 (Wave 5 – Omicron BA1)						
1-4y	11	11	0	0.0	-40.9, 112.8	0.0
5-14y	9	16	-7	-43.8	-64.1, 6.2	-46.4
15-44y	39	41	-2	-4.9	-26.9, 43.2	-10.7
45-64y	54	56	-2	-3.6	-22.5, 26.7	-43.8
≥65y	105	93	12	12.9	-5.0, 37.9	666.6
All ages*	218	217	1	0.5	-10.4, 15.0	2.2

*All ages excluding infants <1 year old.

Table S3a. **Internal validation for 2019. Excess mortality calculated as the difference between observed and predicted deaths from a baseline model using data from 2010-2018.**

Age group	Deaths observed	Deaths expected	Excess mortality N	%	95% PI	Rate/100,000
1st January 2019 – 31st December 2019						
1-4y	69	60	9	15.0	-4.2, 51.7	26.6
5-14y	67	74	-7	-9.5	-25.6, 20.8	-8.1
15-44y	225	229	-4	-1.7	-12.6, 12.5	-3.7
45-64y	225	260	-35	-13.5	-22.3, -3.2	-116.5
≥65y	481	531	-50	-9.4	-16.2, -0.6	-415.1
All ages*	1067	1154	-87	-7.5	-13.1, -1.2	-32.0

Table S3b. **Internal validation of the baseline mortality model.**
Excess mortality was calculated as the difference between observed and predicted deaths where the predictions for each year (from 2010-2018) in turn were obtained by running the baseline model with that year excluded.

Age	Deaths		Excess mortality			
**group**	**observed**	**expected**	**N**	**%**	**95% PI**	**Rate/100,000**
1st January 2010 – 31st December 2010						
1-4y	91	98	-7	-7.1	-23.9, 17.5	-19.2
5-14y	74	60	14	23.3	-5.1, 64.7	18.0
15-44y	286	257	29	11.3	-0.4, 26.3	30.9
45-64y	270	282	-12	-4.3	-13.9, 6.5	-50.1
≥65y	423	410	13	3.2	-6.6, 13.0	154.4
All ages*	1144	1107	37	3.3	-2.9, 8.8	15.4
1st January 2011 – 31st December 2011						
1-4y	93	89	4	4.5	-16.6, 33.9	11.1
5-14y	59	70	-11	-15.7	-31.8, 9.3	-13.8
15-44y	256	267	-11	-4.1	-14.7, 8.3	-11.5
45-64y	249	279	-30	-10.8	-21.6, 0.5	-124.1
≥65y	433	431	2	0.5	-7.4, 13.0	22.6
All ages*	1090	1136	-46	-4	-10.5, 2.5	-18.8
1st January 2012 – 31st December 2012						
1-4y	89	89	0	0	-17.6, 28.1	0.0
5-14y	54	72	-18	-25	-38.7, 0.0	-22.1
15-44y	285	263	22	8.4	-3.7, 23.7	22.0
45-64y	275	266	9	3.4	-6.8, 13.2	36.5
≥65y	470	427	43	10.1	1.8, 19.8	474.8
All ages*	1173	1117	56	5	-0.7, 10.8	22.2
1st January 2013 – 31st December 2013						
1-4y	81	84	-3	-3.6	-22.2, 24.6	-8.2
5-14y	66	70	-4	-5.7	-23.3, 22.2	-4.8
15-44y	228	266	-38	-14.3	-23.5, -2.7	-37.4
45-64y	311	259	52	20.1	6.1, 33.5	205.8
≥65y	414	447	-33	-7.4	-14.9, 3.7	-353.9
All ages*	1100	1126	-26	-2.3	-8.8, 3.5	-10.2
1st January 2014 – 31st December 2014						
1-4y	88	78	10	12.8	-5.4, 43.2	28.0
5-14y	100	68	32	47.1	17.6, 94.3	38.0
15-44y	252	259	-7	-2.7	-14.1, 10.0	-6.7
45-64y	253	269	-16	-5.9	-15.1, 3.7	-61.0
≥65y	415	460	-45	-9.8	-16.8, -0.6	-463.6
All ages*	1108	1134	-26	-2.3	-8.4, 3.4	-10.0
1st January 2015 – 31st December 2015						
1-4y	88	74	14	18.9	0.0, 53.1	39.2

Age group	Deaths observed	Deaths expected	Excess mortality N	%	95% PI	Rate/100,000
5-14y	78	70	8	11.4	-9.3, 50.2	9.5
15-44y	254	251	3	1.2	-9.1, 15.0	2.9
45-64y	269	265	4	1.5	-8.5, 13.0	14.8
≥65y	475	482	-7	-1.5	-8.9, 10.8	-67.4
All ages*	1164	1142	22	1.9	-3.2, 9.1	8.4
1st January 2016 – 31st December 2016						
1-4y	51	75	-24	-32	-42.7, -14.2	-69.1
5-14y	56	75	-19	-25.3	-38.8, -3.4	-22.4
15-44y	246	244	2	0.8	-10.2, 17.4	1.9
45-64y	263	262	1	0.4	-11.1, 12.6	3.6
≥65y	494	497	-3	-0.6	-9.5, 8.9	-27.6
All ages*	1110	1153	-43	-3.7	-10.2, 2.2	-16.4
1st January 2017 – 31st December 2017						
1-4y	59	69	-10	-14.5	-27.2, 12.4	-29.3
5-14y	68	74	-6	-8.1	-24.0, 20.4	-7.0
15-44y	244	236	8	3.4	-7.9, 18.2	7.5
45-64y	252	263	-11	-4.2	-13.1, 4.6	-39.0
≥65y	564	484	80	16.5	6.6, 27.8	715.9
All ages*	1187	1126	61	5.4	-0.8, 10.8	23.0
1st January 2018 – 31st December 2018						
1-4y	73	60	13	21.7	1.4, 58.9	37.6
5-14y	76	72	4	5.6	-14.6, 42.4	4.6
15-44y	239	237	2	0.8	-8.4, 15.0	1.8
45-64y	256	265	-9	-3.4	-14.8, 6.2	-30.7
≥65y	494	538	-44	-8.2	-14.7, 1.8	-376.9
All ages*	1138	1172	-34	-2.9	-7.8, 2.6	-12.5

Table S4. **Excess deaths from 1st January 2020 to 16th April 2022 in Kilifi HDSS residents aged ≥1y, pre-pandemic and by wave analysis. Estimates derived from a model with an additional temperature covariate.** The model with the temperature covariate did not have a significantly better fit than the model without the covariate (Likelihood Ratio Test p-value = 0.0945). This suggest that there is no evidence of an association between temperature and mortality in this setting.

Age group	Deaths observed	Deaths expected	Excess mortality N	%	95% PI	Rate/100,000
1st January 2020 – 31st March 2020 (pre-pandemic period)						
1-4y	15	13	2	15.4	-31.8, 133.0	24.8
5-14y	22	17	5	29.4	-8.3, 120.0	23.7
15-44y	63	55	8	14.5	-11.9, 63.8	30.4
45-64y	71	65	6	9.2	-6.6, 51.3	80.1
≥65y	141	120	21	17.5	-6.0, 49.5	692.7
All ages*	312	270	42	15.6	3.3, 27.4	63.6
1st April 2020 – 4th October 2020 (Wave 1 - wild type)						
1-4y	31	30	1	3.3	-21.1, 89.3	6.2
5-14y	46	36	10	27.8	-7.1, 70.0	23.5
15-44y	124	108	16	14.8	-3.3, 42.3	29.8
45-64y	111	133	-22	-16.5	-29.1, -1.5	-142.2
≥65y	257	294	-37	-12.6	-22.3, -1.4	-584.1
All ages*	569	601	-32	-5.3	-13.9, 3.4	-23.8
5th October 2020 – 14th February 2021 (Wave 2 - wild type)						
1-4y	17	17	0	0.0	-34.7, 62.0	0.0
5-14y	20	26	-6	-23.1	-47.1, 9.3	-20.0
15-44y	79	77	2	2.6	-12.6, 33.2	5.2
45-64y	88	87	1	1.1	-15.6, 23.2	9.0
≥65y	200	185	15	8.1	-4.2, 25.5	326.8
All ages*	404	392	12	3.1	-7.2, 11.8	12.6
15th February 2021 – 4th June 2021 (Wave 3 – Beta-Alpha)						
1-4y	20	15	5	33.3	-12.8, 126.1	53.9
5-14y	21	17	4	23.5	-18.9, 138.9	15.9
15-44y	61	65	-4	-6.2	-20.4, 23.6	-12.2
45-64y	75	79	-4	-5.1	-19.5, 8.6	-43.0
≥65y	179	159	20	12.6	-2.2, 29.9	529.8
All ages*	356	335	21	6.3	-3.8, 15.3	26.1
5th June 2021 – 11th December 2021 (Wave 4 - Delta)						
1-4y	20	24	-4	-16.7	-44.6, 30.1	-24.5
5-14y	19	34	-15	-44.1	-54.7, -23.9	-34.4
15-44y	102	108	-6	-5.6	-17.5, 15.5	-10.4
45-64y	156	125	31	24.8	8.5, 47.5	187.6
≥65y	414	309	105	34.0	20.6, 52.5	1531.1
All ages*	711	600	111	18.5	11.0, 27.2	78.7
12th December 2021 – 16th April 2022 (Wave 5 – Omicron BA1)						
1-4y	19	18	1	5.6	-29.9, 51.8	9.0
5-14y	15	26	-11	-42.3	-60.1, -6.3	-37.1
15-44y	71	78	-7	-9.0	-25.3, 12.3	-17.7
45-64y	95	93	2	2.2	-14.7, 26.2	17.8
≥65y	207	179	28	15.6	0.3, 33.5	610.2
All ages*	407	394	13	3.3	-7.4, 11.0	13.5
1st April 2020 – 16th April 2022 (Waves 1-5)						
1-4y	107	104	3	2.9	-16.3, 22.7	4.7
5-14y	121	141	-20	-14.2	-28.3, 0.7	-11.7
15-44y	437	435	2	0.5	-8.7, 14.1	0.9

45-64y	525	517	8	1.5	-6.0, 10.4	12.6
≥65y	1257	1126	131	11.6	6.2, 19.8	501.1
All ages*	2447	2323	124	5.3	2.6, 9.5	22.7
1st January 2020 – 31st December 2021						
1-4y	107	102	5	4.9	-14.1, 28.2	8.0
5-14y	130	137	-7	-5.1	-20.0, 12.1	-4.2
15-44y	442	424	18	4.2	-5.4, 18.8	8.4
45-64y	519	503	16	3.2	-4.3, 13.1	26.0
≥65y	1243	1095	148	13.5	7.8, 21.7	585.0
All ages*	2441	2261	180	8.0	5.4, 12.6	33.9

Table S5. **Distribution of Kilifi HDSS respondents by period of fieldwork.**

	% of events reported by members within the household		
Year	deaths	out-migrations	enumerations
2010	83	89	98
2011	80	86	97
2012	78	86	98
2013	81	84	98
2014	72	84	96
2015	72	84	96
2016	73	90	97
2017	80	87	98
2018	75	85	97
2019	71	86	96
2020 (Jan - Mar)	78	87	97
2020 (Oct-Dec)	79	81	95
2021	81	84	96
2022 (Jan 1 – May 5)	72	84	96

Table S6. **Age-adjusted 7-month risk of death in cohorts of residents selected on 23rd March each year from 2010-2021**

	All ages		<65 years		≥65 years	
**Cohort year**	**Population**	**7-month mortality risk (%)**	**Population**	**7-month mortality risk (%)**	**Population**	**7-month mortality risk (%)**
2010	248,095	0.44	240,016	0.27	8,079	4.79
2011	253,851	0.35	245,177	0.22	8,674	3.71
2012	258,706	0.36	249,780	0.22	8,926	3.98
2013	266,809	0.35	257,740	0.23	9,069	3.65
2014	271,235	0.34	261,718	0.22	9,517	3.42
2015	275,538	0.35	265,589	0.23	9,949	3.69
2016	278,585	0.32	267,661	0.20	10,924	3.42
2017	285,035	0.38	273,950	0.23	11,085	4.56
2018	289,863	0.31	278,163	0.20	11,700	3.33
2019	296,009	0.28	284,049	0.17	11,960	3.06
2020	300,671	0.30	288,008	0.19	12,663	3.20
2021	300,860	0.32	287,702	0.18	13,158	4.03

Table S7. **Additional VA questions for identification of possible COVID-19 deaths**

ID	Question	Responses
id10482	Was there any diagnosis by a health professional of COVID-19?	yes; no; don't know; refused to answer
id10483	Did he/she have a recent test by a health professional for COVID-19?	yes; no; don't know; refused to answer
id10484	What was the result?	positive; negative; unclear; don't know; refused to answer
id10485	Did he/she suffer from extreme fatigue?	yes; no; don't know; refused to answer
id10486	Did he/she experience a new loss, change or decreased sense of smell or taste?	yes; no; don't know; refused to answer
id10487	In the two weeks before death, did he/she live with, visit, or care for someone who had any COVID-19 symptoms, or a positive COVID-19 test?	yes; no; don't know; refused to answer

Table S8. **Exact dates of re-enumeration rounds, COVID-19 waves and excess mortality analysis periods as illustrated in Figure 1.** Excess mortality analysis windows start 2 weeks after the start date of respective waves, except for wave 1 where the excess mortality starts 2 weeks after the first case was identified. Dates reported as *dd/mm/yyyy.*

Event	Start date	End date
**Re-enumeration rounds**		
Round 48	03/01/2020	11/01/2021
Round 49	12/01/2021	03/05/2021
Round 50	04/05/2021	23/09/2021
Round 51	24/09/2021	14/01/2022
Round 52	15/01/2022	05/05/2022
Round 53	06/05/2022	15/09/2022
**Wave duration**		
Wave 1 (wild-type)	15/04/2020	19/09/2020
Wave 2 (wild-type)	20/09/2020	30/01/2021
Wave 3 (Beta-Alpha)	31/01/2021	20/05/2021
Wave 4 (Delta)	21/05/2021	26/11/2021
Wave 5 (Omicron BA1)	27/11/2021	02/04/2022
**Excess mortality analysis window**		
Analysis 1 (wave 1)	01/04/2020	04/10/2020
Analysis 2 (wave 2)	05/10/2020	14/02/2021
Analysis 3 (wave 3)	15/02/2021	04/06/2021
Analysis 4 (wave 4)	05/06/2021	11/12/2021
Analysis 5 (wave 5)	12/12/2021	16/04/2022

Reference

1 Blangiardo, M. *et al.* Estimating weekly excess mortality at sub-national level in Italy during the COVID-19 pandemic. *PloS one* **15**, e0240286 (2020).

2 Copernicus Climate Change Service (C3S). *ERA5: Fifth generation of ECMWF atmospheric reanalyses of the global climate.*

3 Koen Hufkens and Reto Stauffer and Elio Campitelli. The ecwmfr package: an interface to ECMWF API endpoints.

4 Duarte-Neto, A. N. *et al.* Rapid Mortality Surveillance of COVID-19 Using Verbal Autopsy. *Int J Public Health* **66**, 1604249 (2021)
